# Pharmacokinetic and pharmacodynamic principles: unique considerations for optimal design of neonatal clinical trials

**DOI:** 10.3389/fped.2023.1345969

**Published:** 2024-01-12

**Authors:** Cindy Hoi Ting Yeung, Ruud H. J. Verstegen, Rachel Greenberg, Tamorah Rae Lewis

**Affiliations:** ^1^ Division of Clinical Pharmacology and Toxicology, The Hospital for Sick Children, Toronto, ON, Canada; ^2^ Department of Pediatrics, University of Toronto, Toronto, ON, Canada; ^3^ Duke Clinical Research Institute, Durham, NC, United States; ^4^ Department of Pediatrics, Duke University School of Medicine, Durham, NC, United States

**Keywords:** neonate, clinical trial, pharmacokinetic, pharmacogenetic, dose, pharmacodynamic

## Abstract

Core clinical pharmacology principles must be considered when designing and executing neonatal clinical trials. In this review, the authors discuss important aspects of drug dose selection, pharmacokinetics, pharmacogenetics and pharmacodynamics that stakeholders may consider when undertaking a neonatal or infant clinical trial.

## 1 Introduction

Neonates and infants are commonly exposed to medications that lack sufficient dose finding, pharmacokinetic, safety and efficacy studies (1, 2). Clinical trials, Phase 1–4, are the primary mechanisms through which drugs are adequately studied and labeled in adult populations. Unfortunately, since medications are available off-label, they are routinely introduced into the NICU and other care settings without rigorous data to support dosing and other key issues like pharmacokinetic variability, therapeutic window, and adverse event profile (3). For medications to be studied to a regulatory standard that supports drug labelling, the neonatal research community, funders, drug developers and families must work together to improve the way we currently design and execute neonatal clinical trials.

Neonates are a uniquely important population because this patient population has highly variable physiology, as neonatologists witness in clinical practice every day. Factors that can affect optimal drug dose selection, formulation, drug pharmacokinetics, safety and response are numerous. For example, there is a ten fold weight variation between extreme preterms (450 g) and macrosomic term neonates (4,500 g). There are developmental differences related to gestational age at birth (22 weeks to 42 weeks) and chronological age. Within the Neonatal Intensive Care Unit (NICU) (and within clinical trials), there are populations with extreme differences in organ function such as hypoxemic injury with therapeutic hypothermia, congential heart disease requiring cardipulmonary bypass, and infants with severe sepsis and systemic inflammation. While extrapolation of clinical pharmacology knowledge from adults to older children is often feasible, there are major differences in neonatal physiology that limit extrapolation from adults and older children. For example, limited drug metabolism capacity in neonates does not allow for simple linear size-based predictions in drug clearance, as seen with acetaminophen (4) and caffeine (5). Neonates also have increased vulnerability to side effects not seen in older populations, such as chloramphenicol and grey baby syndrome due to lack of drug glucuronidation (6) and fatal gasping syndrome from benzyl alcohol exposure due to lack of alcohol dehydrogenase capacity (7). Given these stark differences and risks, pharmacology principles are important in the neonatal population.

The goal of this review is to bring a clinical pharmacology lens to neonatal clinical trial design and execution. Clinical pharmacology is the medical specialty that seeks to understand pharmacokinetics (how drugs enter and are processed and eliminated in the body) and pharmacodynamics (the effect that drugs and metabolites have within different organ systems) (8). In addition, clinical pharmacologists seek to understand key sources of variability in pharmacokinetic and pharmacodynamics, such as stage of development or genetic variability. Clinical pharmacology expertise is concentrated at regulatory agencies (FDA, EMA) and within the pharmaceutical industry, so often when investigator-initiated clinical trials in neonates and infants are designed, key pharmacology principles are not considered. In this review, we will provide guidance and examples of important clinical pharmacology principles that can improve neonatal clinical trial design and execution.

Prior to clinical trial design and funding, researchers must consider core pharmacology concepts. These include factors related to pharmacokinetics such as drug absorption, drug metabolism, drug distribution to sites and action and drug excretion. All of these components of pharmacokinetics also have variability (9), and it is incumbent of researchers to understand these sources of variability. For pharmacodynamics, researchers must understand the drug site of action, the required exposure of the drug for clinical effect, and possible safety concerns that may vary among individuals. Published literature about the drug of interest in other patient populations and regulatory documents about the drug can serve as key sources of information. Table 1 provides a list of key resources to find important pharmacokinetic and pharmacodynamic information about drugs.

**Table 1:** Information sources for clinical pharmacology data to be used in neonatal clinical trial design.

Type of information	Potential source		Website
General clinical pharmacology trial considerations for neonates	Regulatory Guidance Documents	Effectiveness, safety, or dose-finding studies in neonates involve assessing clinical pharmacology information, such as information regarding a product's pharmacokinetics (PK) and pharmacodynamics (PD) to inform dose selection and individualization.	https://www.fda.gov/regulatory-information/search-fda-guidance-documents/general-clinical-pharmacology-considerations-neonatal-studies-drugs-and-biological-products-guidance
What is known about drug metabolism?	Regulatory Review Documents	Clinical pharmacology biopharmaceutics reviews cover topics such as receptor effects, dose-response relations, metabolic pathways, pharmacokinetics and how these are affected by demographic factors, comorbid disease, food, and other drugs	www.fda.gov/drugsatfda
What is known about pharmacogenetics?	PharmGKB Pediatric Dashboard	The Pediatric Annotations Dashboard is an entry point to view pediatric annotations on PharmGKB, including PGx guideline annotations, FDA label annotations, variant and clinical annotations, and automated annotations derived from the published literature.	https://www.pharmgkb.org/pediatric/dashboard

## 2 Important aspects of drug interventions to consider when designing neonatal clinical trials

### 2.1 Drug exposure

• *Do we know how the administered dose relates to plasma drug concentrations and target tissue concentrations?*
• *Do we know what concentrations are associated with efficacy and toxicity?*

**Figure 1:**
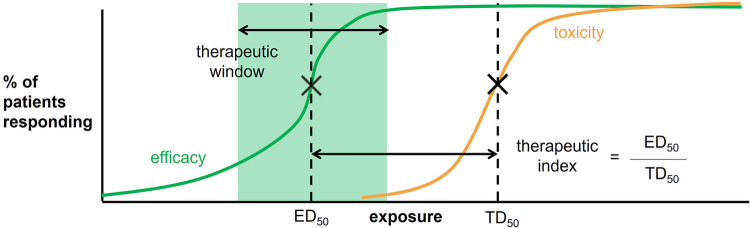
Therapeutic window and therapeutic index are defined according to the percent of patient responding to the drug as a function of an exposure measure (e.g., plasma drug concentrations or AUC). The therapeutic window is highlighted in green. The therapeutic index is defined as the dose required to produce a response in 50% of the population (ED_50_) divided by the dose required to produce a toxic effect in 50% of the population (TD_50_).

To improve our study of drug response in neonates, an understanding of the dose-exposure-response relationship of a drug is necessary. Drug exposure is defined as the drug levels achieved in the body and are typically targeted to attain desired drug tissue concentrations at the site of action and responses of efficacy and safety (10). The drug exposure target is also referred to as the therapeutic window, which is the range of drug exposures that provide sufficient therapeutic response without significant toxic effects (Figure 1). A similar measure that can also be used includes the therapeutic index, which is the ratio between the dose expected to demonstrate some adverse effect (e.g., dose required to produce a toxic effect in 50% of the population) and the dose required for therapeutic effects (e.g., the dose required to produce a therapeutic effect in 50% of the population) (Figure 1). In clinical trials, an intervention of interest is usually compared with standard of care or placebo to assess improvement (or non-inferiority) in efficacy and safety. Therefore, it is useful to identify the drug exposure target at the outset to drive dosing schemes for ensuring that exposure levels are situated around the therapeutic window to make a proper comparison between interventions.

A therapeutic window is typically determined during the stages of drug discovery and development (11). However, since few clinical trials involve neonates and infants in these processes, therapeutic windows specific to these populations are often unspecified. Thus, neonatal and infant drug exposure targets are mainly derived from other pediatric populations and exposure-response studies in neonates and infants conducted after drug development. In both methods, there are two key steps: (1) identifying the appropriate exposure metric in neonates and infants for a given drug (i.e., AUC, C_max_), and (2) identifying the appropriate numeric target within the metric defined in the first step.

For the first step, options for drug exposure metrics can include the area under the curve (AUC), the maximum concentration (Cmax), the minimum concentration (Cmin), and the average concentration at steady state (Cavg,ss). These metrics are further described in Box 1. The choice for the appropriate drug exposure metric can be performed through several methods (12). These approaches can be broadly categorized as extrapolation from other pediatric groups or using exposure-response data in neonates and infants. In the extrapolation approach, it may be reasonable to use a metric applied in other pediatric groups to neonates and infants. For example, AUC_0−6 h_ is commonly used as an exposure metric for intravenous busulfan as a chemotherapeutic agent in children. In neonates and infants, this metric has also been adopted and deemed appropriate through extensive studies in infants (13). The second category of approaches to defining an exposure metric consist of a common strategy to produce estimates of different drug exposure metrics (as identified in Box 1) for each neonate and infant, and choosing the metric most strongly correlated with a clinical outcome or toxicity measure of interest. There are several methods to producing these exposure metric estimates, including noncompartmental analysis, population pharmacokinetics (popPK) modeling, and systematic reviews. In the noncompartmental analysis method, a typical pharmacokinetic study is performed on a cohort of neonates and infants administered the drug of interest (14). The dense pharmacokinetic samples are then used to derive exposure metrics with noncompartmental analysis. The next approach involves popPK modeling (15). PopPK models are mathematical models created to describe the pharmacokinetics and pharmacodynamics of drugs and study sources of variability in exposure and response (discussed further in the Dose Selection section below). In this workflow, only a few sparse samples are needed from each neonate and infant to create the popPK model and output estimated exposure metrics per individual. Finally, in the presence of limited clinical trials in neonates and infants, a systematic review can be performed to compile pharmacokinetic-pharmacodynamic studies in the literature that have identified exposure metrics in this population (16). This approach combines all available exposure metric data to produce a larger sample size.

BOX 1 **Examples of common drug exposure metrics**

**AUC:** Area Under the Curve. AUC is measured by taking the area under the plasma-concentration time profile within a defined time interval (the definite integral). Time intervals can be stated between 0 and infinity (0–∞) or a defined time point (0–τ).

**Cmax:** The concentration of drug at the peak of the concentration-time profile.

**Cmin:** The trough (nadir) of drug concentration on the concentration-time profile.

**Cavg,ss:** Average concentration at steady state (usually attained after 5 half-lives).

Once the appropriate exposure metric has been identified, the next step is to define the appropriate numeric target within the chosen metric. The definition of specific exposure targets utilizes approaches that are a follow-up to those outlined in the first step. Similarly, the first category of methods consists of extrapolating from other pediatric populations. Using the intravenous busulfan example, the exact exposure target or window for children has been defined as an AUC_0–6 h_ of 900–1,500 µmol·min/L (13). This exposure window can also be used in neonates and infants, which was confirmed in a study in this population with the target demonstrating an AUC_0–6 h_ of 900 µmol·min/L as most appropriate. Although this extrapolation approach would be suitable for intravenous busulfan, important caveats on using this method must be explained. First, this approach assumes that the pharmacokinetics of the drug is highly predictable with linear kinetics, as is the case for intravenous busulfan. Often, the dose-exposure-response relationship of adults and children can differ from neonates and infants. In the same way, neonatal and infant pharmacokinetics and pharmacodynamics can vastly differ from children which calls for infant population-specific exposure targets. For example, a standard C_min_ is commonly used as a vancomycin exposure metric in children and adults, though, a systematic review by Mejias-Trueba et al. (17) reported on the immense variability of C_min_ targets in neonates. Second, drugs with indications exclusive to neonates and infants will be disadvantaged since target exposures in children may not exist. Third, an appropriate exposure metric and target may be dependent on the dosing regimen and administration. For instance, use of a range of C_max_ for a drug administered as a single dose may be suitable in children; however, if the drug is administered as a multiple dose for neonates or infants, a C_max_ range is no longer a useful exposure metric indicator since it will be greatly influenced by drug accumulation.

Based on the disadvantages on relying on extrapolations from other pediatric populations to define the target exposure window, it is often optimal to perform the second category of methods that rely on pharmacokinetic samples from neonates and infants. Again, these methods are a follow-up to those described in the first step of defining the most suitable exposure metric. Once the exposure metric has been chosen, it is then used to define the percentage of patients responding to the drug, thereby creating a relationship for both the efficacy and toxicity outcomes to define the therapeutic window and index (Figure 1). The following cases present examples of the different variations of this approach as introduced in the first step. Yeh et al. (18) demonstrated that plasma indomethacin levels 12 h after the dose correlated with efficacy (patent ductus arteriosus closure) and toxicity outcomes (hyponatremia), and a 600 ng/ml was associated with 50% odds of treatment success, but more renal side effects (18). In a similar approach, the authors could have also used noncompartmental analysis to define further exposure metrics to identify exposure targets. Hirt et al. (19) demonstrate using sparse ibuprofen plasma concentrations to build a popPK model to identify AUC after the first dose of ibuprofen <600 mg·h/L and the cumulated AUC for three daily doses of ibuprofen <900 mg·h/L, as highly predictive of patent ductus arteriosus closure efficacy in neonates. As described in a review by Tyson et al. (20), Greenberg et al. (21) and Ericson et al. (22) present examples of a further approach that uses exposure-response data to identify therapeutic windows. The authors conducted systematic reviews for select antiepileptic and immunosuppressant drugs to retrieve and summarize their pharmacokinetics, efficacy, and adverse reactions data to define exposure targets.

### 2.2 Dose selection

• *What data are informing our dose selection for this clinical trial?*
• *What are the knowledge gaps that we need to address to feel confident in our dose selection?*

Alternative methods for dose selection in neonates and infants have been employed in lieu of clinical trials in these populations. Historically, the main approach to dose selection in neonates and infants has been empirical or scaled. In scaled dosing, adult doses are modified by a specific factor to suggest appropriate pediatric doses. Common scaling methods include Clark's Rule (based on weight), Young's Rule (based on age), weight-based dosing (adjusting by mg/kg), body surface area dosing (adjusting by mg/m^2^), and allometric scaling based on weight ratio to the power of 0.75. These methods assume that the pediatric drug target exposures are equal to adults (i.e., pharmacokinetics and pharmacodynamics are the same), and therefore aim to maintain an adult exposure. Although these scaling approaches account for growth, they do not account for the maturation of relevant processes. As a result, dose selection based on scaling often overestimates doses in neonates and infants as they experience rapid maturational changes and variability in pharmacokinetics. Huang et al. (23) provide an example demonstrating the importance of including the maturation of key drug metabolizing enzymes of valproic acid, CYP2C9 and UGT2B7, on dose selection. The authors show that except for Clark's rule, most conventional scaling methods would lead to under- or over-dosing in neonates and infants. For instance, in comparison with physiologically-based pharmacokinetic (PBPK) model approach, which is a tool that uses a mechanistic understanding of drug disposition in the body (including enzyme ontogeny) to predict drug exposures (discussed further in the paragraph below), body surface dosing of valproic acid in infants would lead to almost an inappropriate 2-fold increase in C_max_ (23).

In recent years, in silico modelling approaches have been increasingly used for dose selection (24, 25). In silico pharmacology (also known as computational therapeutics, computational pharmacology) is a rapidly growing area that encompasses techniques for using software to capture, analyse and integrate biological and medical data to aid in drug development and research. Two common modelling approaches that have been mentioned previously are popPK and PBPK modelling. A popPK model is empiric and built using subject data for model building which resembles a “top down” approach. In the context of neonates and infants, data are gathered from these populations treated with a drug and used to create the popPK model. In contrast, PBPK models are mechanistic and built from the “bottom up”. PBPK models are mathematical representations of drug disposition in the body that require information on the physicochemical properties and absorption, distribution, metabolism and excretion of the drug, and anatomy and physiology of the organism. PBPK models can be used to model drug pharmacokinetics in a population in a data rich environment (e.g., adults) to predict the pharmacokinetics in other populations (e.g., neonates and infants). PopPK models use collected neonatal and infant pharmacokinetics data to describe their drug exposures and predict doses. Pediatric PBPK models rely on changing the anatomy and physiology from adults to neonates and infants, and at different ages, accounting for growth and maturation of relevant processes to predict neonatal and infant pharmacokinetics (26). Neonatal and infant pharmacokinetics data are not necessary in pediatric PBPK modelling, and where available, are used for confirmatory purposes. PopPK and pediatric PBPK models can be preferred over the scaling methods to dose selection due to their ability to account for covariates that can influence drug pharmacokinetics and maturation factors based on the actual mechanisms responsible, respectively. In both modelling strategies, the dose-exposure relationship is elucidated so that given a target exposure, the needed dose can be estimated with the models. As a result, these modelling approaches have been used to guide dose selection and influence drug labelling. Examples include use of popPK modelling for dapaglifozin and pediatric PBPK modelling for trazodone to help inform European Medicines Agency drug labelling for pediatric use to treat type 2 diabetes mellitus and pediatric insomnia, respectively (27, 28). Moreover, popPK and pediatric PBPK models are gaining traction for use in model-informed precision dosing which provides a more personalized dose selection in neonates and infants. Among hospitalized neonates and children, achievement of therapeutic vancomycin trough concentrations was improved after implementation of a pharmacokinetic model-guided precision dosing approach (29).

### 2.3 Drug formulation

• *Can we reliably dose the drug accurately based on the formulation we have chosen?*
• *What are the excipients in the formulation and what do we know of their safety?*

For a clinical trial to be successful, consideration around the accurate administration of the study drug is an important step. Drug formulation encompasses issues such as matrix of drug (liquid vs. solid form), compounding required vs. commercial formulation available, the stability of the dose form, excipients (additives) in the formulation, and the ability to measure accurately and dose accurately the weight-based doses that will be used in the clinical trial (30). For neonatal trials, these considerations are not trivial and require early and ongoing collaboration with research pharmacists to understand and plan accordingly.

Unique considerations in neonates include parenteral and enteral dosing volume. In preterm and term infants, reliable ability to enterally dose large volumes (>3 ml) are limited, and if an infant is inpatient and has IV fluids and medications, the total volume of parenteral study drug can impact fluid balance. In addition, inpatient neonates may be receiving enteral medications through different feeding tubes, and the interaction of the study drug with enteral tubing can affect the actual dose delivered in neonates and infants (31). Since neonates require very small doses of drugs, the actual ability to measure the drug volume reliably and dose it reliably becomes an issue. In a study developing guidelines for accurate measurement of small volume products in syringes, the authors found that dose measurement error was more likely when less than 20% of the capacity of the syringe was used. Dose measurement error ranged from 1.4% to 18.6%, despite syringe manufacturer specification of ±5% accuracy, suggesting proper syringe use technique as a major factor in small-volume measurements (32). Drug volumes less than 0.1 ml have been associated in the literature with administration errors attributed to calculation, dilution and equipment (33). Developing neonates with limited toxin clearance capacity may also be vulnerable to effects of harmful excipients commonly used in drug formulations (34). All components of the drug, not just the active ingredient, must be considered for safe drug dosing in clinical trials. And although infants may be commonly exposed to potentially harmful excipients (ethanol, propylene glycol, benzyl alcohol) during clinical dosing of medications, the risk introduced under the auspices of research are viewed differently.

Interestingly, the stringency of information required for drug products used in research may be more than if the same drug were used in clinical care, so when designing a clinical trial, physicians may be overwhelmed with the amount of drug specific information that is unknown. Working closely with pharmacists and clinical pharmacologists can allow for a structured and streamlined collection of formulation-specific information that will strengthen the internal validity of trial results.

## 3 Clinical pharmacology concepts for neonatal clinical trial design

• *If the drug will be dosed enterally, what is known about bioavailability?*
• *What are the paths of drug metabolism and elimination, and do they differ by patient age?*

Clinical pharmacokinetic processes include drug absorption, distribution, metabolism and excretion. In neonates, each of these processes may have unique development aspects that require careful consideration in designing clinical trials. The topic of neonatal clinical pharmacology has been extensively reviewed in prior publications (35, 36), but in this section we will highlight a few critical factors that can impact clinical trial design.

The knowledge of drug absorption and bioavailability that is described in older populations may not be pertinent to neonates and young infants (37). For example, any food effects on drug dissolution and absorption can be magnified in infants who rarely have stomachs empty of human milk or formula feeds. Acid buffering by milk feeds leads neonates and infants to have, on average, a higher gastric pH which may change absorption of some drugs, but neonates are known to reach near-adult levels of gastric acidity between feeds. Gastric emptying and intestinal transit time are important determinants of drugs delivery to the mucosal surface of the small intestine. Gastric emptying time can be delayed by immature peristalsis in preterm infants and can also be influenced by type of milk fed (fortified vs. unfortified breastmilk) (38). A combination of drug transporters (i.e., MDR1) and intestinal drug metabolizing enzymes (i.e., CYP3A4) can impact enteral drug absorption. When designing a neonatal clinical trial, compiling all available information on drug absorption and bioavailability can help researchers choose a data-informed dosing regimen.

**Figure 2:**
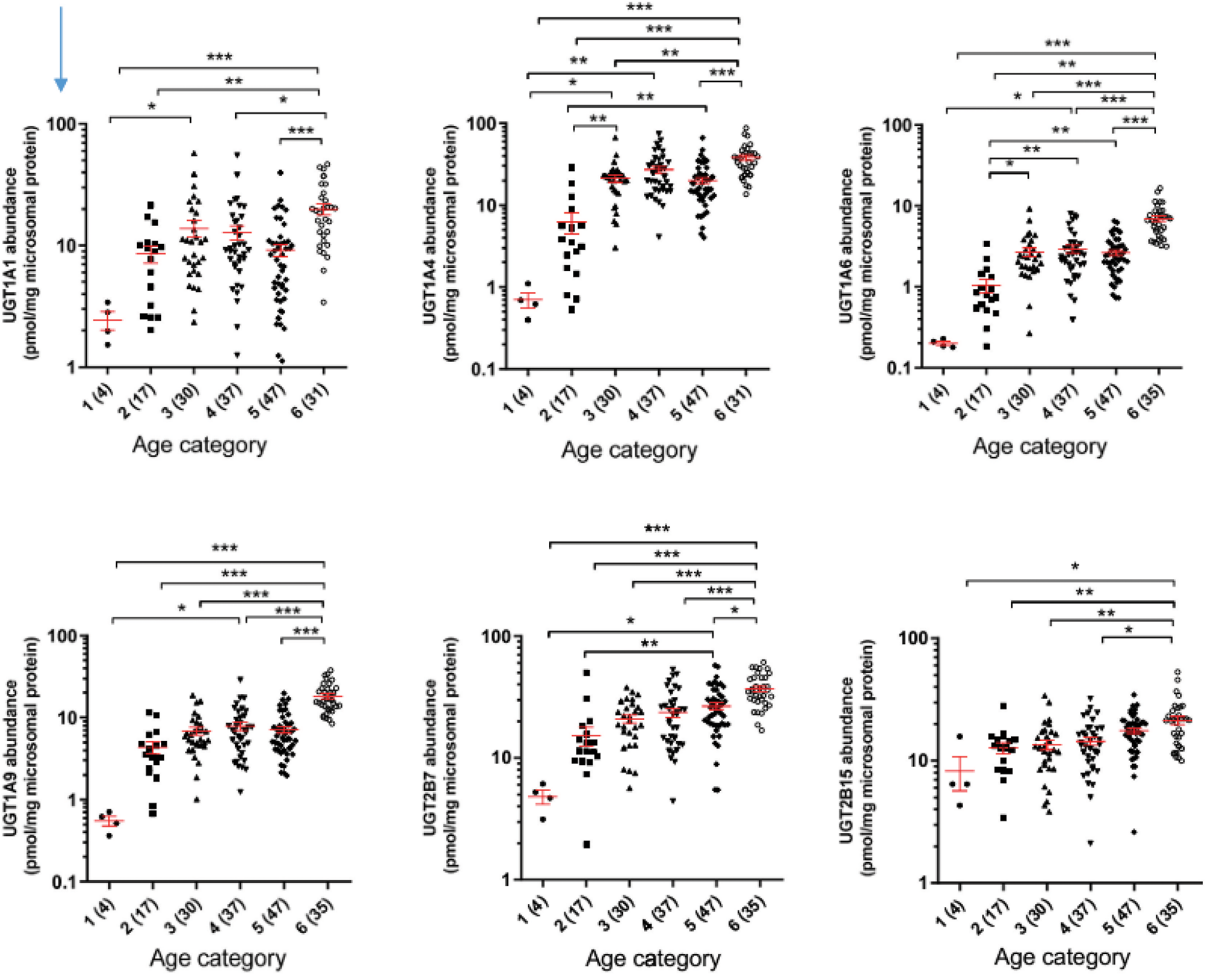
Developmental trajectories of six major uridine diphosphate-glucuronosyltransferases from human liver samples. Age categories on horizontal axis are (1) = 0–27 days, (2) 28 to 364 days, (3) 1-6 years, (4) 6–12 years, (5) 12–18 years, (6) >18 years. From Bhatt et al. (40).

Ontogeny (development) of drug-metabolizing enzymes and drug transporters is an important source of interindividual variability in pharmacokinetics for infants and children. For example, an infant who is born at 23 weeks' gestation and treated with a drug at 4 weeks of age likely metabolizes that drug differently than an infant of similar birth gestational age who is started on the same drug at 8 weeks of age. An infant born at 23 weeks of age who is 4 weeks old metabolizes very differently from a 27 week infant who has just been born. So, both postmenstrual and postnatal age have impacts. Although dosing based on weight in part corrects for developmental changes, weight-based dosing often does not capture other development processes fully. The levels of protein expression of key hepatic drug-metabolizing enzymes are generally lower at birth (with the exception of CYP3A7) and increase with advancing age (39). Each enzyme and enzyme class has slightly different trajectories of development. For the uridine diphosphate-glucuronosyltransferase (UGT) enzymes, extensive proteomic profiling has revealed that each UGT has a distinct ontogenic profile, but the expression is generally low at birth and increases within the first few months of age (40), as displayed in Figure 2.

### 3.1 Measuring and describing pharmacokinetic information

• *What is known about the pharmacokinetics of this drug in neonates and where are key knowledge gaps?*
• *How can we design our study to fill these knowledge gaps?*

Over the years, multiple factors and developments have created improved opportunities to collect neonatal and infant pharmacokinetic samples for clinical trials. Specifically, in the NICU, several different types of clinical sampling allow for drug quantification in pharmacokinetic studies. First, neonates in the NICU sometimes have indwelling arterial lines that make it possible to collect dedicated pharmacokinetic samples (e.g., multiple timed sample points from a single dosing interval, given assay volume requirement and size-based blood drawing limits). Second, blood samples are commonly drawn for clinical labs in the NICU, which offers another opportunity to retain a portion of the sample for drug measurement or “scavenging” these samples after they have been used in the hospital laboratory. The leftover plasma can be collected and used for drug quantification. Importantly, the research team must work closely with a quantitative chemist who will optimize the drug (and metabolite) assay for performance in very small plasma volumes (<100 ul). Third, dried blood spots (DBS) can be collected through heel stick blood draws, which are the standard method to obtain blood from infants for routine hospital laboratory tests. When using the DBS approach, hematocrit levels should be recorded in each infant, and plasma samples should also be drawn from a few infants. Hematocrit levels and matched plasma samples can be used to convert drug DBS concentrations to plasma concentrations (41, 42). An advantage of collecting drug concentrations through DBS is that each pharmacokinetic samples only requires 20–30 ul of blood, making more frequent sampling feasible even with limited neonatal blood volumes. More information on blood volume requirements can be found in a tutorial by Shakhnovich et al. (43).

Another matrix that is important to collect in neonates and infants is urine. Drug concentrations in urine assist in the partitioning of renal clearance for understanding how the drug is cleared in these populations. Additionally, metabolites of certain drugs can be measured in urine samples which provide information on drug metabolism (44). In the NICU, urine can easily be collected with a cotton ball placed in the diaper. The volume of urine can be ascertained by weighing the diaper before and after urination, and these cumulative urine volumes can be used to quantify drug and metabolite excretion.

Encouragingly, in silico modeling methods have made it possible to only require a few plasma or DBS samples from each neonate or infant (e.g., 2–3 samples) to gain an understanding of drug pharmacokinetics. In traditional methods, dense pharmacokinetic sampling is required per individual to properly characterize models accurately for predictions. However, popPK can incorporate sparse pharmacokinetic samples from each individual and involve diverse patient populations which can reduce the burden of sampling per neonate or infant and improve model generalizability. To leverage the contribution of a diverse patient population, it is important to collect demographic variables such as gestational age, postnatal age, sex, birthweight, weight, pharmacogenotypes, concomitant medications, and disease state. These variables can serve as covariates in popPK models to describe some of the wide variability observed in this patient population. It is key to note that a popPK model can only accurately predict the pharmacokinetics in the population it was built upon. Thus, if the intention is to understand the pharmacokinetic in extremely preterm infants, a sufficient number of infants <28 weeks would need to be enrolled in the pharmacokinetic study.

One of the challenges unique to neonates and infants is ascertaining appropriately timed samples for adequate pharmacokinetic analysis. Since many of the samples are opportunistic and there are restrictions to blood draw volumes, it can be difficult to retrieve the needed samples for adequately characterizing pharmacokinetic profiles. An example of target sample times that are optimal to build a popPK model for neonates administered morphine as an intravenous infusion and several boluses, and an eventual switch to enteral dosing is depicted in Box 2.

BOX 2 **Example of ideal plasma sample times drawn from neonates for morphine popPK development**

• Start of the first IV infusion, before reaching steady state
• Immediately before and after IV infusion dosing changes
• Immediately before and after (to capture C_max_) an IV bolus
• A later time point from an IV bolus (e.g., 24 h post dose to capture the elimination phase)
• When switching from IV to enteral dosing (compare steady-state concentrations)
• Immediately before and after (to capture C_max_) an enteral dose

### 3.2 Pharmacodynamics and drug response biomarkers

• *What is the drug target and the developmental trajectory of the drug target?*
• *What are biomarkers (outside of primary endpoint) that we can collect to strengthen our understanding of drug response physiology?*

Pharmacodynamics encompasses all the biochemical, physiological and clinical effects of a medication on the body. One of the main paradigms in pharmacology is that the therapeutic effect is directly related to the drug concentration at the site of action. From this perspective, it is understandable that variations in pharmacokinetics can result in widely varying drug concentrations, and thus differences in the response to an identical dose. However, even if groups of individuals obtain identical drug exposures at the site of action, marked variations can be observed where some groups experience no effect and others experience toxicity.

**Figure 3:**
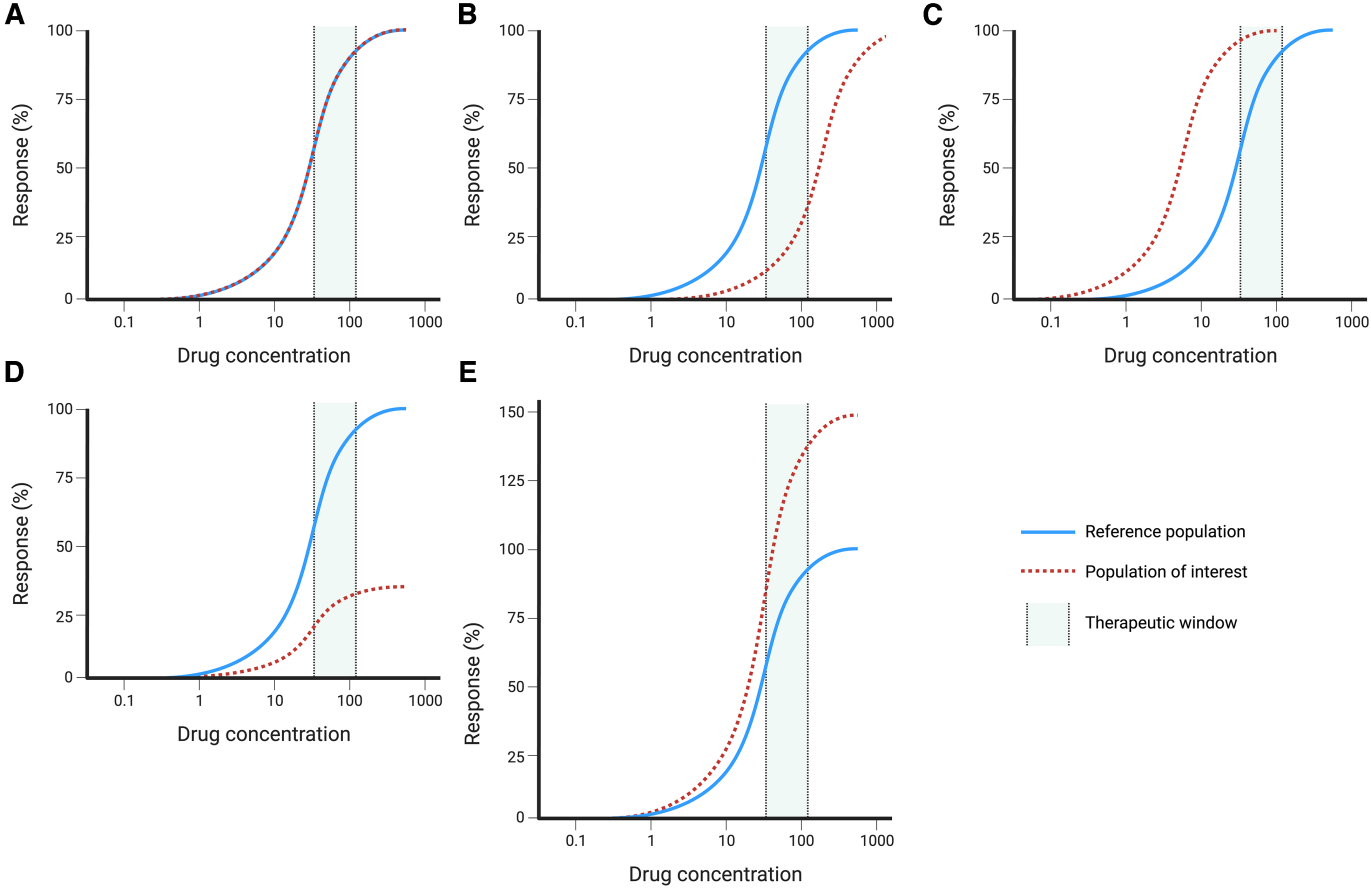
Hypothetical dose-response curves with reference population (solid blue line) and population of interest (dashed red line): (**A**) no changes, (**B**) reduced potency, (**C**) increased potency, (**D**) decreased efficacy, (**E**) increased efficacy. Created with BioRender.com.

The different scenarios are best illustrated by hypothetical exposure-response curves that compare the average relationship between exposure (or dose) and the response to those seen in specific populations (Figure 3). These curves illustrate the difference between potency and efficacy. In some instances, the potency is reduced which means that an increased dose is required to achieve the same efficacy (Figure 1B). Similarly, the opposite can be present where an increased potency needs lower dosing to avoid toxicity (Figure 1C). When comparing the response, the maximum attainable effect may be reduced or increased (Figures 1D,E), which affects drug efficacy and the likelihood of developing toxicity.

**Figure 4:**
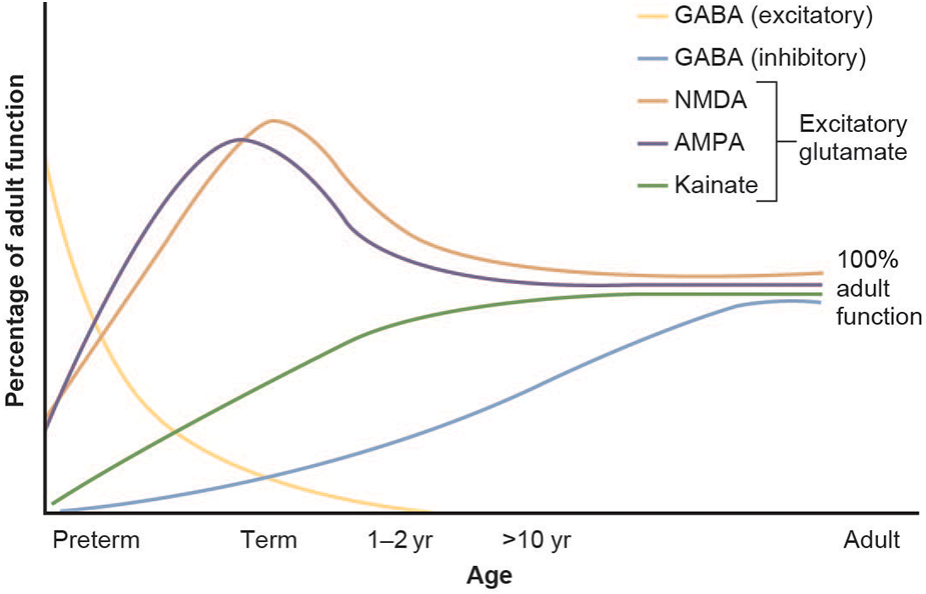
Developmental changes in glutamate and GABA receptor function in the brain. Figure adapted from Rhakade and Jensen (45).

There are numerous factors that influence the individual's response and explain why neonates can have treatment responses that are much different from those seen in older children and adults. Upon drug-target binding (e.g., extracellular receptors, enzymes, etc.), most medications inhibit or stimulate downstream intracellular processes. It is therefore understandable that if variations exist in the structure or expression of these drug target sites, medications may have a different affinity that is lower or higher, and result in diminished or increased effect. In the context of newborns, it is known that the expression of many drug target sites depends on the pregnancy term and postnatal age. For example, GABA and glutamate receptor expression are known to have age-specific developmental patterns (Figure 4) (45).

Interactions with other medications, environmental factors (e.g., diet, toxins) or endogenous proteins (e.g., hormones, cytokines) may all result in altered binding of medications to the drug target, as well as cause diminished or enhanced downstream effects. Similarly, in neonates with co-morbidities, such as infections and surgical congenital heart defects, their response to medications may be different than anticipated. Inflammation (during infection) is known to affect drug transport and drug metabolizing enzyme expression and function (46, 47). In congenital heart disease, cardiopulmonary bypass can cause significant alterations in pharmacokinetics in the post-operative period (48), so special consideration of pharmacology principles is warranted.

When studying the effect of pharmacotherapeutic agents on neonates, it is crucial to consider the optimal clinical and biomarker outcomes. In some cases, it is relatively uncomplicated to determine the drug effect in real-time, for example when addressing sedation (e.g., midazolam), blood pressure (e.g., dopamine) or urinary output (e.g., furosemide). However, the complexity of the underlying physiological system affected by the medication may cause challenges. For example, the immune system consists of many cellular and humoral components that undergo rapid development in neonates. When medications impact the immune system, the intended or unintended effects may be delayed, difficult to quantify, and affected by other variables such as gestational age, post-natal age or infections.

### 3.3 Pharmacogenetics

• *For the drug under study, have there been pharmacogenetic investigations in other patient populations?*
• *Based on the stage of development of study cohort, and their expression of the relevant proteins, do we expect to see variability due to pharmacogenetics?*

Clinicians can often predict how an infant will respond to a specific medication and adjust the treatment (e.g., dose) based on available population studies and clinical experience. However, some neonates exhibit an unexpected response or develop an unusual adverse drug reaction. These variations in treatment response may be related to gestational age, ontogeny, and underlying diseases, however, there is an increasing awareness that the individual's genetics influence how one responds to pharmacotherapy.

Pharmacogenomics is the field that studies the impact of genetic variations on the pharmacokinetics and pharmacodynamics of medications in a population. These studies may be untargeted [i.e., genome-wide association study (GWAS)] where no or limited restrictions are placed on the analysis of the genomic data. This type of research often requires relatively large populations to identify genes or variants significantly associated with a specific treatment response. Alternatively, studies may focus on specific genetic variants that have a known effect on protein function or receptor expression and an established role in drug metabolism or response in other populations. This approach may potentially reduce the size of the required study population.

Pharmacogenomic studies in neonates are complicated by the influence of gestational age and postnatal age on drug metabolism and response, but recently groups have compiled available pharmacogenetic knowledge in neonates (49). As discussed before, children are born with low enzyme activity for most enzymes that then increase according to an enzyme-specific pattern (50). Importantly, our genetics do not only affect the maximum enzyme activity achieved at maturation, but it already influences enzyme activity in the neonatal period. For example, a study on tramadol metabolism in neonates showed that increased CYP2D6 enzyme activity differed between normal and ultrarapid metabolizers, independent of gestational age (51).

There are examples of pharmacogenetic variability having an impact on clinical at the bedside in the NICU. As recently published (52), neonates in the NICU had rapid point-of-care (26 min turnaround time) for a genetic variant in gene *MT-RNR1* which prediscposes to aminoglycoside-induced ototoxicity. In this multi-site trial, they identified infants who carried the risk variant and these infants avoided aminoglycoside antibiotics during their care. This is an example where pharmacogenetic research findings originally established in other patient populations (53) can inform clinical research studies in the neonatatal population.

Over the past decades, a large amount of pharmacogenomic data has been generated that has enhanced our understanding of how an individual's response to medication is influenced by their genetics. PharmGKB (Pharmacogenomics KnowledgeBase; www.pharmgkb.org) is an online resource that collects, curates and disseminates knowledge about clinically actionable gene-drug associations and genotype-phenotype relationships. It contains data from exploratory studies to large clinical trials and includes available clinical practice guidelines. Recently, they have added a pediatric specific page that highlights knowledge from pediatric studies. Clinicians and researchers can use this resource to identify if their drug of interest has any identified pharmacogenetic associations in other patient populations. For example, if a neonatologist was designing a clinical trial with morphine as a study drug, there are many morphine pharmacogenetic studies described in this resource that could be used to inform study design.

When pharmacogenomic data is used for clinical decision-making, and testing is targeted to identify specific genetic variants, we often refer to this as pharmacogenetic testing. Regulatory agencies, such as the Food and Drug Administration (FDA) and the European Medicines Agency (EMA), increasingly include pharmacogenomic data with clinical guidance. However, most detailed and practical information is provided by professional organizations. The Clinical Pharmacogenetics Implementation Consortium (CPIC) and Canadian Pharmacogenomics Network for Drug Safety (CPNDS) in North America, and the Dutch Pharmacogenetic Working Group (DPWG) in Europe have been continually translating pharmacogenomic evidence into clinical practice guidelines that provide recommendations for healthcare providers on how pharmacogenetic test results can be applied in the care of individual patients.

Currently, there are nearly 400 medications with pharmacogenetic data included in the product monograph and almost 200 that have dedicated pharmacogenetic clinical practice guidelines (pharmgkb.org). Due to the limited available data in neonates, no guidelines are available for this population (49). However, it is important to be aware of medications that have clear clinical indications in neonates and have pharmacogenetic guidance in adults (Table 2). Incorporating pharmacogenotyping into clinical trial design can help advance knowledge in whether genetic variation is important for neonatal drug metabolism and response.

**Table 2:** Medications used in neonatal care with pharmacogenetic guidelines for older children and adults.

Drug class	Drug name	Gene(s)
Proton pump inhibitors	Omeprazole	CYP2C19
	Pantoprazole	
Anticoagulation	Warfarin	CYP2C9, VKORC1, CYP4F2
Non-steroidal anti-inflammatory drugs (NSAIDs)	Ibuprofen	CYP2C9
Platelet inhibitors	Clopidogrel	CYP2C19
Antiepileptics	(Fos)phenytoin	CYP2C9
Halogenated anesthetics	Desflurane	CACNA1S, RYR1
	Enflurane	
	Halothane	
	Isoflurane	
	Sevoflurane	
Anti-emetics	Ondansetron	CYP2D6
Antibiotics	Amikacin	MT-RNR1
	Gentamicin	
	Kanamycin	
	Tobramycin	

When designing neonatal clinical trials, assessing the literature for pharmacogenetic associations in other populations is an important step.

## 4 Conclusion

In summary, there are multiple important clinical pharmacology considerations that should be considered during neonatal clinical trial design. Early collaboration with clinical pharmacologists (either within the drug development industry or academia) is encouraged because they have expertise that will complement the neonatal knowledge of clinical trialists.Without adequate consideration of concepts such as goal plasma exposure targets, rational dose selection based on developmental pharmacology, adequacy of drug formulation, measurement of pharmacodynamics and biomarkers, and the potential influence of pharmacogenetics, we miss the opportunity to optimally design our trials and to learn new and important knowledge about disease physiology and drug response. Neonatologists and neonatal clinical trialists play a crucial role in advancing therapeutic options for neonates and infants. A more expansive vision of team science brings all relevant expertise to the clinical trial design table, and will allow for more fulsome pharmacology considerations and optimal trial design.
